# Independent prescribing in the UK: insights from the Department of Health Allied Health Professions Medicines Project team

**DOI:** 10.1186/s13047-023-00641-z

**Published:** 2023-07-04

**Authors:** Ivan Bristow, Catherine Bowen, Nicky Wilson, Alan Borthwick

**Affiliations:** 1 AECC University College, Bournemouth, UK; 2 Centre for the History of Podiatric Medicine, Royal College of Podiatry, London, UK; 3 School of Health Sciences, Faculty of Environmental and Life Sciences, University of Southampton, Southampton, UK; 4 NIHR, Clinical Senate of the Royal College of Podiatry, London, UK; 5 King’s College Hospital NHS Foundation Trust, London, UK; 6 School of Clinical Sciences, Queensland University of Technology, Brisbane, Australia; 7 School of Health and Human Sciences, Southern Cross University, Lismore, Australia

**Keywords:** Department of health, Allied health professions, Medicines, Commission on human medicines, Medicines and healthcare products regulatory agency, Health and care professions council, Patients, Workforce, Service redesign

## Abstract

**Background:**

The UK medicines legislation was amended ten years ago (2013) to allow podiatrists and physiotherapists independent prescribing rights, the first of the allied health professions to do so. Non-medical prescribing formed one part of a broader policy agenda promoting role flexibility in response to the challenge of an ageing population and the need to maintain effective health provision in the face of a contracting workforce.

**Aim:**

The aim of this study was to outline the experiences of the Department of Health AHP medicines project board team in working towards independent prescribing for podiatry and physiotherapy, with a particular focus on the challenges encountered.

**Methods:**

In depth, open-ended interviews were conducted with eight of the core members of the project team, drawn from those individuals who served throughout the duration of the project (2010–2013). Included were the former Department of Health Chief and Deputy Chief Allied Health Professions Officers; the Department of Health Engagement and Communications Officer; representatives of the Health and Care Professions Council; the Medicines and Healthcare products Regulatory Agency; the Council of Deans of Health; the Royal College of Podiatry and the Chartered Society of Physiotherapy (The team also included the representative of the Allied Health Professions Federation. However, as that representative is also a researcher in this study, he has recused himself from any role as a participant.). Data were transcribed and subject to a thematic analysis.

**Results:**

A complex picture of the project emerged revealing a range of obstacles and challenges, including inter-professional role boundary tensions and negative prior assumptions about the two professions. Success hinged upon the adoption of a dual strategy involving submission of a robust case of need focused on patient benefit coupled with the careful management of professional expectations. Underpinning theory from the sociology of the professions offers a supportive explanatory framework for understanding the relationships between the various stakeholders involved.

**Conclusions:**

Ultimately, success depended upon aligning the project aims with healthcare policy through a clear focus on patient benefit. Balancing competing professional and policy demands through a continual emphasis on improved patient care laid the foundations for future projects by other allied health professions.

## Background

In 1999 the ‘Crown Report’ was published, recommending the extension of rights to prescribe medicines to a number of different nursing and allied health professions [1]. Most notable were those suggested as early candidates for independent prescribing,^1^ including optometrists, tissue viability and family planning nurses, specialist podiatrists and specialist (extended scope) physiotherapists [1]. The report highlighted the potential to improve health outcomes, enhance patient experience and ensure better use of resources [1]. It also resonated firmly with other contemporary policy initiatives addressing the impact of demographic changes (an ageing population) through workforce flexibility [2–9]. By 2008 nursing, pharmacy and optometry had attained independent prescribing rights [4]. In order to provide a response on behalf of the allied health professions, the Chief Allied Health Professions Officer (CAHPO) at the Department of Health (DH) initiated a scoping project (2008–2009) to examine the evidence of need for, as well as the feasibility of, allied health professions prescribing. Its conclusions recommended a further project to advance the case for podiatry and physiotherapy independent prescribing [10]. In 2010, a new project board convened, and its work culminated in approval by the Commission on Human Medicines (CHM) in 2012, followed by legislative change in 2013.^2^

The aim of this study was to map the multiple stages involved in the process, and to outline the experiences of the project board team as they attempted to navigate each step, with particular emphasis on the challenges and obstacles encountered.

Contextualising these changes within the dynamics of the health division of labour is supported through the use of an explanatory sociological framework for understanding the nature of interprofessional relationships across the health professions [11–14]. Within this framework, inter-professional tensions may be manifest in jurisdictional disputes focused on task domains and role boundaries [15, 16]. Protecting role boundaries forms one part of the process of social closure, in which professions “are seen as limiting access to opportunities to a restricted group of eligibles” [17–19]. The strategies deployed may include the use of credentials, legal / legislative protection of exclusive rights or titles, or the support of powerful elites [15–17]. Given that role boundaries in the UK are seldom immutable, competition for space within a given domain remains a constant challenge for professions [20–24]. Achieving the desired outcome depends upon the degree to which significant others – more powerful professions, the state and the public – can be engaged as resources or persuaded of the case for change [4, 14, 21].

## Methods

The data in this study were derived from a series of in depth, open-ended qualitative interviews with a number of key actors drawn from the Department of Health Allied Health Professions Medicines Project Board, established in 2010 and concluding in 2013.^3^ A purposive, criterion-based^4^ sampling strategy was adopted, in order to obtain data from individuals who had taken part in the project throughout its duration, and which formed the core members of the project board [25]. Each individual was approached via email with an explanation of the proposed project and an invitation to participate. This included the DH Chief and Deputy Chief Allied Health Professions Officers, the DH Communications and Engagement Officer, the representatives of the Chartered Society of Physiotherapy, the Royal College of Podiatry, and the Council of Deans of Health, as well as the policy officers from the Medicines and Healthcare products Regulatory Agency (MHRA) and the Health and Care Professions Council (HCPC).^5^ The interviews were semi-structured, adopting a brief interview guide allowing considerable scope for participant elaboration. Ten years on from the legislative changes, these core members were invited to reflect on the journey, providing insights into the challenges facing the project. All interviews were transcribed verbatim, and subject to a thematic analysis in line with the Braun and Clarke method [26, 27]. Two of the research team undertook the analysis (see author contributions at the end of the paper). Initial familiarisation with the data was undertaken by the two analysts. This comprised a reading and re-reading of the transcripts. The coding stage was then undertaken to identify data related to the study aim. The data was interrogated to identify sub-themes, which, where similar, were collated to form the main themes. Finally, the research team discussed the themes with a view to refining or modifying them in order to reach full agreement. Power relationships were fully considered and central to the analysis, underpinned by the theoretical approaches outlined within the sociology of the professions literature. These address the power issues of professional dominance, jurisdictional disputes, occupational closure strategies and interprofessional tensions and are used to understand the narrative in this paper [11–18].

Data extracts, drawn from in-depth interviews, are therefore substantive, providing information-rich material [25]. Data generated from the interviews were triangulated with available documentary material.

## Results

The data analysis yielded five key themes:

- The complexity of the processes involved
- The challenge of inter-professional rivalry
- The need to prioritise patient benefit over professional ambitions when convincing key audiences (CHM, Health Ministers and professions)
- The challenge of clarifying the skill levels in podiatry and physiotherapy
- Facilitating future bids by other AHP professions.

These themes are presented through a narrative that follows a chronological sequence, guiding the reader through the various stages of the process, starting with the initial scoping project, and culminating in the final submission to the main CHM and its aftermath.

The initial scoping project – designed to ascertain need and establish evidence to support a full project – required funding from within the Department of Health, as well as a team to contribute to the work. The work of establishing the project also revealed early signs of opposition.

*The funding within the Department [of Health] at the time - we had hardly any money in AHPs - was within the Nursing Directorate, that’s where we were at the time…we were being exposed to [the] concern that somehow we were invading their territory, but that was why the scoping study was so important. So, rather than agitate to do work on getting independent prescribing for physios or podiatrists or anyone else, I took a step back. Now, I knew that the professions [AHPs] were resistant to what appeared to be a retrograde step but I knew that there was absolutely no way we were going to get anywhere with a) getting any money, or b) making the case, unless we made the case around patients…* [CAHPO]

*There wasn’t a team then, so there were a lot of telephone calls to try and ascertain what the interest was. The calls were initially with the professional bodies, so if they weren’t interested, then there was no point in following it up. And then we got together a project team. So, it was trying to develop that case of need … And I have to say, you did get the impression there was a lot of scepticism, as to whether any of these other professions would need to use prescribing mechanisms. Ministers… needed to have a good understanding of the professions…we knew that those outside AHP land might not have a really good idea*. [Deputy CAHPO]

*The evidence was a bit sketchy at the time. Those of us who pride ourselves on robust evidence might have been scrambling around for a little robust evidence … But it hadn't been tried and tested. So there was a leap of faith into this* [Council of Deans of Health representative]

One other consideration in establishing a scoping project was the need to demonstrate impartiality when dealing with the aspirations of each of the allied health professions, not all of which sought access to prescribing. By the time it concluded, the scoping project provided sufficient evidence to select those cases considered most likely to succeed.

*As chief allied health professions officer, I had to do it for all the allied health professions. I couldn’t be seen to favour one... The scoping report came out and [recommended] physiotherapy and podiatry and possibly radiography. We had the biggest issue with…OT [occupational therapy], because the professional body at the time said they should be included. But there was no ammunition, there was no evidence [of need], if you like, at the time. Obviously, it’s changed since. But, because we had done all that work, it stood up to scrutiny*. [CAHPO]

*The CAHPO was clear that we needed to scope all of the [allied health] professions, whether or not they were using other mechanisms. Some weren’t interested at all, some had a tiny minority that were interested, and therefore the case of need wasn’t as strong*. [Deputy CAHPO]

Once the scoping project was completed – which took a year [10]—the next challenge was to secure funding to support its recommendations in taking forward work towards physiotherapy and podiatry independent prescribing. Interprofessional tensions complicated matters and delayed progress.

*Resistance to the potential for physiotherapists and podiatrists to prescribe was anticipated from the medical profession but, surprisingly, it also came from pharmacy too…So, I did loads of work with policy leads on areas of care pathways - I needed this third party endorsement just for us to get the money to do the work. And in the end, we got it, but it was pharmacy that took the greatest persuading. So I had to do a massive charm offensive…that it wasn’t about threatening their position, there was [prescribing] work for everyone. I have never thought this was about money [access to funds]. It was always about inter-professional rivalry. *[CAHPO]

*We thought the nurses would be entirely onside with this, but there was a little bit more politics playing out, which we didn’t see coming, which was that nurses were quite happy themselves to get independent prescribing, but when it came to expanding the field to others … they were a little bit less enthusiastic than we expected them to be.* [Communications and Engagement Officer DH]

After DH funding had finally been secured, a project board had to be established to work through the various stages towards a final submission to the CHM. As the first two allied health professions to negotiate this journey, each step in the process presented its own challenge for the board. The initial stages included an engagement exercise, a public consultation exercise and an equality impact assessment. Later, practice guidance documents and a competency framework for prescribing were required, alongside HCPC standards for prescribing and an outline curricular framework document guiding education providers.^6^ Granting rights to ‘new’ prescribing groups like the allied health professions added to the challenge. The engagement and public consultation work required considerable efforts in diplomacy, detail and consistency in communications.

*…what we didn't want to happen was to do a statutory process and find it not supportive, because that might close the door forever. So I distinctly remember it was [named individual] at the MHRA who suggested that we use a stakeholder engagement process as a soft touch first stage to really test the water before having to follow the very strict timetables and process of a formal statutory consultation* [Physiotherapy representative]

*I was tasked with preparing for the consultation, getting everything ready to go to consultation. Almost like every public consultation, you go out for a period of engagement ahead of that, to sound out key organisations like the Royal Colleges... And, as I recall, it was the medical Royal Colleges that were pushing back most. So it was a matter of coming up with a form of words and a set of proposals that they were more likely to agree to whilst still keeping true to the spirit of what we were trying to do. It was the same arguments that we kept hearing coming up, but we knew that we had done a fairly reasonable job because at least we knew what the arguments were at the very outset, even before we launched the public consultation. We’d heard a lot of the medical profession saying that they were concerned about people prescribing outside of their field of expertise. So it was a constant campaign of reassurance...* [Communications and Engagement Officer, DH]

*The problem was … it was like a leap of faith... You could…talk about all the diabetic foot ulcers, of Friday night and not being able to get antibiotics and it’s the weekend and then the ulcer’s breaking down…. But there was no data to say independent prescribing would stop that, so that was tricky with stakeholders* [CAHPO]

One key feature of the public consultation was the enormous response generated from within the physiotherapy and podiatry professions. Whilst hugely supportive, there was concern this might appear somewhat biased, and under-represent other influential stakeholder views – most notably those of the medical authorities. Thus, the engagement exercise proved pivotal in addressing this effect.

*There was overwhelming support for the proposals from the professions. But what about the big guns? So I just said, ‘perhaps when you're discussing the outcome of the consultation, it might be better not to just say, ‘well, everyone in the profession wants that’. It’s not that it's not valid, but it's good to know what others, like the Royal Colleges and other important stakeholders think as well* [MHRA representative]

*In any stakeholder engagement map you map interest level, but also influence level. So, we knew that all the AHPs would be interested, but perhaps not every single [AHP] clinician would be the most influential in the final decision making. The influence is where you come to the medical Royal Colleges… we did need to get them onside because they were a highly influential group…* [Engagement and communications officer, DH]

Perhaps the greatest challenge faced was the introduction of an additional step, which involved preliminary submissions to two sub-groups of the Commission on Human Medicines (one each for physiotherapy and podiatry) prior to the final submission before the main Commission. Although it had been done before to help clarify the case of need for ‘less well known’ professions (such as optometry), here it appeared to cast doubt on the appropriateness of podiatry and physiotherapy prescribing, and nearly led to a rejection of the submissions.

*… we organised having the sub-working groups [CHM sub-committees] to consider the proposals in more detail and then bring their decision or their thoughts back to the full CHM. We'd done it before, and I thought it might be a good idea because this was the first time [for AHPs]… There was less knowledge about podiatrists and physios, and, you know, ‘why would they be prescribing?’. I just felt that if someone could consider these proposals in more detail it would help the process when it went to CHM. We had done something similar with the pharmacists, and for optometrist independent prescribers. So it kind of seemed like a good idea at the time*. [MHRA representative]

*But I think some of the people that we came across had very strongly held views about who should and shouldn't prescribe and what was a good model of regulation and what wasn't. Most people don't understand regulation. That's fine. But where people aren't willing to engage and understand or don't want to engage, to understand, that can be a bit more of a problem. And I think we did come across a bit of that…I think it was quite bruising and trying to explain that just because, for example, it was a different regulator, didn't mean it was any less safe or effective* [HCPC representative]

*…we knew it [might] be antagonistic, we knew that from the [start]. But I knew we had the answers, and we could explain… I believed they wouldn’t be able to say ‘no’ because we had done everything right… we found out their thoughts and feedback and … it was probably the most deflated I ever felt in my career because I genuinely also thought, ‘well that’s it’.* [Podiatry representative]

The negative reception of the two cases by the CHM subgroups prompted the CAHPO and her deputy to seek an audience with the chair of the main Commission, convened after these events but before the final submission.

*…the pre-meetings with the [sub groups] of the Commission on Human Medicines, … went dreadfully wrong…The view was - that was it! … it felt very, very harsh. I [then] met the chair of the CHM in a private meeting. He said enough to indicate that he knew they had overstepped the mark…we should resubmit and it would all be treated very, very differently. I think that this was a bit of a turning point. I think there was a little bit more empathy for our position.* (CAHPO]

*The reason that [CAHPO] and I went to meet with the [CHM] chair was because of the outcome of the meeting with the [sub-group of] CHM, and the reasons that they had given... We went with a fair bit of trepidation, because we were challenging how the decision had been arrived at, which we felt had been pre-determined... I think some of the comments indicated that they had already had some discussion and they were not looking favourably on the case of need.* [Deputy CAHPO]

In response, the chair of the CHM provided guidance in the form of seven key principles that would need to be adopted, although the ultimate outcome was still far from certain. The final submission involved a face to face encounter with the full CHM, comprising a large panel of medical and scientific experts.^7^ A detailed presentation was made, followed by robust questioning.

*I think it was helped that when the proposals went to the full CHM, the chair at the time had put in place a kind of a checklist. It was seven principles to consider. It actually made things a lot more straightforward. When the proposals were considered within that framework it kind of made it easier, too.* [MHRA representative]

*I knew that we had to take a certain approach, and not be defensive about what we were trying to do. It felt like some sort of trial…It was quite daunting. I can also remember feeling, as I was talking, I felt on top of my game, I knew we had it, I knew that … there wasn’t anything we had left out. We’d looked at this from every possible angle…I [also] knew we had one hit at this.* [CAHPO]

The CHM gave its approval following the submission and presentation, and the project team felt the outcome achieved not only tangible benefits to patient care, but the creation of a gateway for other allied health professions to attain independent prescribing.

*I think we shouldn't ignore the fact that it was the trailblazer, and it led to next steps…with my various hats on I continue to talk to the Department of Health…about going forward with the paramedics and so on…so I think it's important just to reinforce the fact that it was this work that was the trailblazer for a lot of fantastic work to follow* [Council of Deans of Health representative]

*…in terms of the other professions, there’s no doubt in my mind that it was…easier for them as a result … they still had to do all the work to justify it, but they knew the things they would have to do to get it through.* [CAHPO]

Equally, it was clear that physiotherapy and podiatry had been ill-understood by the CHM sub-committees, underpinning the apparent reluctance to support the submissions. In that context, use of the collective term ‘allied health professions’ did little to help clarify the distinct roles and contributions of physiotherapy and podiatry, and may even have impeded a clear understanding.

*The conversations I had with leaders of other professions were so full of outdated assumptions, for example, ‘why would physios who massage patients in a back room need to prescribe?’, and ‘[do] podiatrists cutting toe nails need to prescribe? …On the one hand, the collective term [AHP] is useful in terms of exerting influence…but in many ways the drive to use the term to our advantage did and is doing a disservice when it comes to promoting practice in a particular profession as we were trying to do with IP. What is an AHP? Patients and the public might just know or have heard of the individual professions, but really don’t know what an AHP is – nor do Ministers, incidentally* [CAHPO]*.*

## Discussion

The findings clearly convey the range of difficulties encountered by the project team. Fractious inter-professional relations, unflattering assumptions about podiatry and physiotherapy and the sheer complexity of the process were serious complications. Perhaps the main key to success lay in developing a case centred on patient benefits rather than arguing for greater rights for the professions, thus establishing a sound justification which chimed with service need. In turn, this created a model for later bids by therapeutic radiography and paramedics.

Inter-professional rivalry created obstacles and threatened to derail the submission. A reluctance to support change on the part of other professions – medicine, nursing and pharmacy – hindered progress. Neo Weberian perspectives from within the sociology of the professions often highlight the hierarchical power relations within healthcare [14, 20, 21]. Although considered to exert diminished dominance in recent years, medicine is clearly still able to impose a degree of both social and cultural authority, as this study testifies [4, 8, 12]. Abbott’s concept of ‘jurisdictional dispute’ in particular captures the tensions that may arise over contested role boundaries, seemingly at odds with a policy agenda intent on role flexibility but evident in this study nonetheless [15, 16].

Professions, by nature, do not easily cede authority over task domains or agree to blur role boundaries in areas considered central to professional identity [4, 17]. The prescribing of medicines is a core function of medical practice and one previously exclusive to doctors^8^ [4, 28–32]. Indeed, although softening rigid professional boundaries via increased role flexibility is a longstanding government policy objective [7–9], the study data do reflect an underlying resistance to change. These concepts provide a lens through which the events in this study may be viewed and understood, but with one significant corollary. The Department of Health team, led by the CAHPO, understood that the project would not succeed if it prioritised the professionalising ambitions of the professions themselves. Rather, it sought to emphasise improvements in patient care as a guiding motive; an aim consistent with and aligned to government health policy objectives.

## Conclusions

The challenge for the DH AHP Medicines Project team was to fully comply with the requirements necessary to persuade the state (from Ministers of Health to the CHM) of the need, safety and viability of independent prescribing for podiatrists and physiotherapists. Leading the first AHP bid for independent prescribing meant the DH team required a strong case of need that could be justified in the face of robust challenge, as well as align the professions’ ambitions with the wider policy agenda. Its success undoubtedly laid the groundwork for future submissions by other allied health professions.

## Data Availability

Data consisted of interview transcripts which remain in the possession of one of the authors (AB) in password protected files, in line with University of Southampton policy. Internal documents of the Department of Health Project were accessed, but are not available for public access. Public documents, such as the 2008–9 Scoping Project, are available online.

## Abbreviations

CAHPO: Chief Allied Health Professions Officer
CHM: Commission on Human Medicines
MHRA: Medicines and Healthcare products Regulatory Agency
HCPC: Health and Care Professions Council
DH: Department of Health
IP: Independent Prescribing

## Footnotes

1 The Crown Report (1999) suggested two new versions of prescribing; ‘dependent’ and ‘independent’. The former later became known as ‘supplementary’ and represented a form of prescribing dependent upon prior approval of a clinical management plan by a designated physician. Independent prescribing did not require physician approval, allowing the practitioner to make a diagnosis and instigate treatment independently.

2 Human Medicines (Amendment) Regulations (2013) Statutory Instrument 2013, SI No 1855. London: The Stationery Office. URL: http://www.legislation.gov.uk/id/uksi/2013/1855

3 This also included the preceding scoping project, which ran between 2008 and 2009 and which comprised many of the same personnel.

4 A criterion based sampling strategy refers to specific criteria used to select potential participants. In this case the criteria were individuals who had taken part in the project as members of the DH AHP medicine project team, and who were therefore able to provide first-hand experience of the issues concerned in the paper. They were also members who served throughout the duration of the study.

5 The Health and Care Professions Council was known as the Health Professions Council at the time of these events.

6 Securing access to specific lists of controlled drugs was also undertaken, involving a submission to the Advisory Council on the Misuse of Drugs of the Home Office, requiring similar documentation, with changes sought to the Misuse of Drugs legislation. See Fitzpatrick and Borthwick 2022 (ref [28]).

7 The CHM panel comprised primarily physicians/professors of medicine as well as professors of pharmacy, epidemiology and medical statistics, alongside lay representatives.

8 Strictly speaking, exclusive to doctors, dentists and veterinary practitioners (Medical Act 1968).

## References

[1] Department of Health (1999). Final Report of the Review of Prescribing, Supply and Administration of Medicines (Crown Report).

[2] Borthwick A, Nancarrow S, Scriven A (2005). Promoting Health: the Role of the Specialist Podiatrist. Health Promoting Practice.

[3] Nancarrow S, Borthwick A (2005). Dynamic professional boundaries in the healthcare workforce. Sociol Health Illn.

[4] Nancarrow S, Borthwick A (2021). The Allied Health Professions: A Sociological Perspective.

[5] Foot D, Gomez R (2006). Population ageing and sectoral growth: the case of the UK 2006–2026. Oxford Journal of Business and Economics.

[6] Cameron A, Masterson A, Davies C (2003). Reconfiguring the Clinical Workforce. The Future Health Workforce.

[7] Department of Health (2000). A Health Service of all the Talents: developing the NHS workforce.

[8] Boyce RA, Chesters J, Kitto S, Thistlewaite J, Reeves S (2011). Borthwick A, Moran M, and Nancarrow S, Health workforce reform: dynamic shifts in the division of labour and the implications for interprofessional education and practice. Sociology of Interprofessional Health Care Practice: Critical Reflections and Concrete Solutions.

[9] Department of Health (2000). The NHS Plan - A Plan for Investment, A Plan for Reform.

[10] Department of Health (2009). Allied health professions, prescribing and medicines supply scoping project report.

[11] Saks M (2010). Analyzing the Professions: The Case for the neo-Weberian Approach. Comp Sociol.

[12] Saks M (2015). The Professions, State and the Market.

[13] Adams TL (2004). Inter-professional conflict and professionalization: dentistry and dental hygiene in Ontario. Soc Sci Med.

[14] Nancarrow S, Borthwick A, Dent M, Bourgeault I, Kuhlmann E (2016). Interprofessional working for the health professions: From fried eggs to omelettes?. The Routledge Companion to the Professions and Professionalism.

[15] Abbott A (1988). The System of Professions: An Essay on the Division of Expert Labour.

[16] Abbot A, Liljegren A, Saks M (2017). Boundaries of social work or social work of boundaries?. Professions and Metaphors: understanding professions in society.

[17] Saks M (2021). Professions: A Key Idea for Business and Society.

[18] Saks M. Defining a profession: The role of knowledge and expertise. Professions and Professionalism. 2012;2(1).

[19] Agevall O, Social closure: on metaphors, professions and a boa constrictor. In Liljegren A and Saks M (Eds), Professions and Metaphors: understanding professions in society. Abingdon: Routledge. 2017.

[20] Sak M, Liljegren A, Saks M (2017). Regulating the English health professions: zoos, circuses or safari parks?. Professions and Metaphors: understanding professions in society.

[21] Saks M, Dent M, Bourgeault IL, Denis JL, Kuhlmann E (2016). Professions and power: a review of theories of professions and power. The Routledge Companion to the Professions and Professionalism.

[22] Drennan VM, Gabe J, Halter M, de Lasignan S, Levinson R (2017). Physician associates in primary healthcare in England: a challenge to professional boundaries?. Soc Sci Med.

[23] Brydges M, Dunn JR, Agarwal G, Tavares W (2022). At odds: How intraprofessional conflict and stratification has stalled the Ontario paramedic professionalization project. Journal of Professions and Professionalization.

[24] McDermott I, Astbury J, Jacobs S, Willis S, Hindi A, Seston E, Schaftheutle E (2023). To be or not to be: the identity work of pharmacists as clinicians. Sociol Health Illn.

[25] Fetterman DM. Ethnography: Step-by-step. London: Sage publications; 2019.

[26] Braun V, Clarke V (2006). Using thematic analysis in psychology. Qual Res Psychol.

[27] Braun V, Clarke V (2021). One Size Fits all What Counts as Quality Practice in (Reflexive) thematic Analysis. Qual Res Psychol..

[28] Fitzpatrick TJ, Borthwick AM (2022). A decade of independent prescribing in the UK: a review of progress. Journal of Foot and Ankle Research.

[29] Borthwick A, Short A, Nancarrow S, Boyce R (2010). Non-Medical Prescribing in Australasia and the UK: The Case of Podiatry. Journal of Foot and Ankle Research.

[30] Borthwick A, Kilmartin T, Freeman C, Wilson N, Franklin P (2017). Allied Health Prescribing. Non-Medical Prescribing in the UK.

[31] Borthwick A, Nolan P, Bradley E (2008). Professions allied to medicine and prescribing. Non-Medical Prescribing - Multi-disciplinary Perspectives.

[32] Maher A, Borthwick A (2022). An audit of the prescription and supply of medicines by podiatric surgery teams in the UK. Journal of Prescribing Practice.

